# Knowledge, Attitude, and Practice of Contraception Methods Among Female Undergraduates in Dodoma, Tanzania

**DOI:** 10.7759/cureus.4362

**Published:** 2019-04-02

**Authors:** Waheeda Shokat K Kara, Magreth Benedicto, Jing Mao

**Affiliations:** 1 Nursing Psychiatry, Tongji Medical College, Huazhong University of Science and Technology, Wuhan, CHN; 2 Psychology, St. John's University of Tanzania, Dodoma, TZA; 3 Nursing, Tongji Medical College, Huazhong University of Sciences and Technology, Wuhan, CHN

**Keywords:** knowledge, attitude, practice, contraception, female university students

## Abstract

Introduction

Contraception is regarded as an important preventive measure of unintended pregnancies and sexually transmitted diseases, including human immunodeficiency virus infection and acquired immune deficiency syndrome (HIV/AIDS), among youths. This study aimed to assess the knowledge, attitude, and practice of contraception among female undergraduates in Dodoma, Tanzania.

Methodology

A cross-sectional study was conducted among 347 female undergraduates of St John’s University, Dodoma. Descriptive statistics were used for data analysis. Statistical analysis was done using Epi-Info version 7.2.2.6 (Centers for Disease Control and Prevention, Atlanta, Georgia. A p-value of less than 0.05 was considered statistically significant.

Results

The mean (±SD) age of participants was 27.4 (± 5.7). The majority (96%) of the participants were aware of contraception. Awareness of contraception was significantly associated with the age (p<0.0001), marital status (p<0.00001), and religion of the participating students (p=0.02). Slightly less than half (47.4%) of the students reported having ever used at least one type of contraception while feeling embarrassed to buy or ask for contraception (64.6%) and differing religious beliefs (32.3%) were among the reasons reported by students for not using contraception.

Conclusion

Despite the relatively low utilization of contraception, the majority of the participants had knowledge of contraception. This calls for efforts to advocate the effective utilization of reproductive and sexual health services among youths.

## Introduction

Almost 20% of the Tanzanian population is between 15 and 24 years of age [1]. This group constitutes the majority of youths who join universities in Tanzania. Although the group is the most sexually active, girls in the group are faced with more challenges in accessing contraceptives than are married women due to the stigma attached to their sexual activities before marriage. This poses the risk of unwanted and teen pregnancies and unsafe abortions [2]. The latter is a significant public health concern in many developing countries, with the most recent publication showing global annual incidence estimates that suggest that 25.1 million women had undergone unsafe abortions between 2010 and 2014 [3].

Despite being freely available at health facilities throughout the country, the utilization of contraception among students, especially females, remains low, with increased rates of unplanned pregnancies being reported [4]. Previous reports in Tanzania have shown the utilization of contraception among secondary school students to be between 11.8% and 40.0% despite students exhibiting a relatively high knowledge of contraception [5-8]. In the general population, the contraception uptake rate has been reported to vary from 19.0% to 34.4% [4,8].

The correct use of contraception can prevent unintended pregnancies, unsafe abortions, and sexually transmitted infections, including HIV [9]. Unwanted pregnancies among students of higher learning institutions create a major public health problem, particularly in developing countries, and may jeopardize students' learning and potential careers. Globally, several studies have reported on the knowledge, attitude, and practice toward the use of contraception among various groups of students [10-14] but little is known about the current knowledge, attitude, and practice toward the use of contraception among young females in this study setting.

Most previous studies have concentrated mainly on primary and secondary school girls, and only a few have addressed the problem of the low utilization of contraception among university students. Further, to the best of our knowledge, no study has been conducted in this study area to assess the knowledge, attitude, and practice of methods of contraception among female university students. This study, therefore, aimed to assess the knowledge, attitude, and practice of contraception methods among female undergraduate students in Dodoma, Tanzania.

## Materials and methods

Study design, setting, and population

This was a cross-sectional descriptive study conducted among undergraduate university students in Dodoma city, Tanzania. The study population consisted of female undergraduate students at the main campus of St John’s University of Tanzania. This is one of the largest universities in the city and is located about five km from the city center. The university offers both undergraduate and postgraduate programs to about 5000 students.

Sampling and sample size

The study site was randomly selected from a list of universities located in Dodoma. A sample size of 347 students was calculated using the Kish, Leslie formula [15] based on the previous prevalence of users of contraception (34.4%) reported in a study conducted at another university in eastern Tanzania [5], with a 95% confidence interval and a ±5% degree of precision.

Inclusion and exclusion criteria

The study included all first to fourth-year, full-time, registered, female undergraduate students of all age groups. Another inclusion criterion was a willingness to participate in the study. Exclusion criteria included an unwillingness to participate and being a part-time or distance learning student.

Data collection

Students who consented to participate were interviewed using pre-tested, self-administered structured questionnaires. The collected information included socio-demographic characteristics, knowledge of students on modern contraceptives, students' attitude toward the use of modern contraceptives, and students' practices toward the use of contraception. Dependent variables included knowledge, attitude, and practice of contraception, whereas independent variables were age, year of study, marital status, program of study, and source of information about contraception.

Data analysis

Statistical analyses were done using Epi-Info version 7.2.2.6 (Centers for Disease Control and Prevention, Atlanta, USA). Continuous data were expressed as means ± standard deviation (SD) and categorical data as percentages. The chi-square tests were used to determine the association between categorical variables. A p-value of less than 0.05 was considered statistically significant.

Ethical considerations

Informed written consent was obtained from each participant. The study protocol was collaboratively approved by the Institutional Review Board of Tongji Medical College, Huazhong University of Science and Technology, and the University Research Internal Ethical Committee of St John's University of Tanzania.

## Results

Socio-demographic characteristics of respondents

A total of 347 students were recruited in the study, with a response rate of 100%. The mean ± SD age of participants was 27.4±5.7 years. Approximately 68.0% of the participants were aged between 21 and 30 years, 80.4% were single, and 40.6% were first-year students. Table 1 summarizes the socio-demographic characteristics of the study participants.

**Table 1:** Socio-demographic characteristics of the study participants, n=347 Abbreviations: FOCB, Faculty of Commerce and Business Studies; FAHE, Faculty of Humanities and Education; FANAS, Faculty of Natural and Applied Sciences

Categorical variables	Frequency, n (%)
Age	
15-20	25(7.2)
21-30	235(67.7)
31-40	77(22.2)
41-50	10(2.9)
Marital status	
Single	279(80.4)
Married	62(17.8)
Cohabiting	2(0.6)
Divorced	4(1.2)
Religion	
Christian	210(60.5)
Muslim	134(38.6)
Others	3(0.9)
Faculty	
FOCB	132(38.0)
FAHE	114(32.9)
FANAS	101(29.1)
Year of study	
First year	141(40.6)
Second year	123(35.4)
Third year	83(24.0)

Knowledge of contraceptive methods

The majority of the participants, 333 (96.0%), were aware of contraception. Knowledge of methods of contraception was assessed by scoring the responses of participants on the various methods of contraceptives they know. If a participant mentioned none or only one correct method of contraception, she was regarded as having poor knowledge, whereas good knowledge was when a participant mentioned two or more correct methods. Of the participants who were aware of contraception, 83.5% mentioned oral contraceptives as one of the methods of contraception and 45.7% mentioned mass media as their main source of information regarding contraception. Of those who were aware of contraception, 92.2% stated that contraception prevents unwanted pregnancies. Table 2 shows the knowledge of students about contraception.

**Table 2:** Knowledge of students regarding contraception

Categorical variables	Number, n(%)
Awareness on contraception (n=347)	
Aware	333 (96.0)
Not aware	14(4.0)
Knowledge of methods of contraception (n=333)	
Good	326(97.9)
Poor	7(2.1)
Known methods of contraception (n= 333)	
Pills	278(83.5)
Condoms	293(88.0)
Injectable contraceptives	199(59.8)
Natural methods	131(39.3)
Implants	124(37.2)
Loop	99(29.7)
Tubal ligation	87(26.1)
Vasectomy	85(25.5)
Known reasons for using contraception (n=333)	
Prevention of unwanted pregnancy	307(92.2)
Prevention of sexually transmitted infections	285(85.6)
Means of family planning	302(90.7)
Sources of information about contraception (n=333)	
Mass media	152(45.7)
Friends	76(22.8)
Health care workers	69(20.7)
School teachers	26(7.8)
Parents	10(3.0)

We used the chi-square test to determine the association between the awareness of contraception and socio-demographic characteristics of the study participants. We found a significant association between the awareness of contraception, the age of participants (p<0.0001), marital status (p<0.00001), and the religion of the students (p=0.02). Table 3 shows the association between the awareness of students of contraception and their socio-demographic characteristics.

**Table 3:** Association between awareness of contraception and socio-demographic characteristics, n= 347 Abbreviations: FOCB, Faculty of Commerce and Business Studies; FAHE, Faculty of Humanities and Education; FANAS, Faculty of Natural and Applied Sciences

Categorical variables	Aware, n (%)	Not aware, n(%)	p-value
Age			<0.0001
15-20	21(84.0)	4(16.0)	
21-30	232(98.7)	3(1.3)	
31-40	72(93.5)	5(6.5)	
41-50	8(80.0)	2(20.0)	
Marital status			<0.00001
Single	270(96.8)	9(3.2)	
Married	61(98.4)	1(1.6)	
Cohabiting	1(50.0)	1(50.0)	
Divorced	1(25.0)	3(75.0)	
Religion			0.02
Christian	204(97.1)	6(2.9)	
Muslim	127(94.8)	7(5.2)	
Others	2(66.7)	1(33.3)	
Faculty			0.36
FOCB	129(97.7)	3(2.3)	
FAHE	109(95.6)	5(4.4)	
FANAS	95(94.1)	6(5.9)	
Year of study			0.14
First year	132 (93.6)	9(6.4)	
Second year	121(98.4)	2(1.6)	
Third year	80(98.8)	3(1.2)	

Attitudes of participants toward contraception

Table 4provides a detail of the attitudes of students toward the use of contraception methods. About 65.8% of the participants responded that contraceptives tend to reduce sex drive while 32.7% of the participants said condoms can slip off during sexual intercourse. About half (50.8%) of the students reported that it was more desirable to use contraception than to have an abortion.

**Table 4:** Attitudes of students toward the use of contraception, n = 333

Attitudes toward contraception	Number, n(%)
Contraception is beneficial	271(81.4)
Contraception reduces sex drive	219(65.8)
Condoms can slip off during sexual intercourse	109(32.7)
Contraception better than abortion	169(50.8)
Will use contraception in future	41(12.3)

Practice of contraception methods

About 47.4% of the students who had knowledge of contraception had never used any method of contraception in their lifetime. Among the reasons for not using condoms were: contraception is against religious beliefs (32.3%) and a feeling of embarrassment while buying or asking for contraception at the facilities (64.6%). Oral contraceptives were the most commonly used method of contraception (54.9%) while pharmacies were the most common source of contraception (53.1%). Table 5 shows the practices of study participants toward the use of contraception.

**Table 5:** Practices of students toward the use of contraception

Variables	Number, n(%)
Ever used contraception (n = 333)	
Yes	175(52.6)
No	158(47.3)
Reasons for using contraception (n = 175)	
To prevent unwanted pregnancy	138(78.9)
To prevent sexually transmitted infections	150(85.7)
For family planning	59(33.7)
Others	22(12.6)
Reasons for not using contraception (n = 158)	
Against religious beliefs	51(32.3)
Reduces sexual pleasure	47(29.7)
Causes cancer	12(7.6)
Causes weight gain	21(13.3)
Not readily available	3(1.9)
My partner disapproves	46(29.1)
I fear its side effects	43(27.2)
Lacking enough knowledge on how to use	9(5.7)
Embarrassed to buy/ask for them	102(64.6)
Used contraceptive methods (n = 175)	
Pills	96(54.9)
Condoms	58(33.1)
Injectable contraceptives	13(7.4)
Norplants	5(2.9)
Other methods	3(1.7)
Places to get contraception (n = 175)	
Pharmacy	93(53.1)
Nearby government health care facilities	46(26.3)
Private health facilities	8(4.6)
Other facilities/sources	28(16.0)
Ever used emergency contraception (n = 183)	
Yes	69(37.7)
No	114(62.3)
Reasons for not using emergency contraception (n=114)	
No knowledge of emergency contraception	72(63.2)
On other contraception	25(21.9)
Fear of side effects	17(14.9)

We also assessed the awareness, practices, and attitudes of students toward the use of emergency contraception. Of the 333 students who were aware of contraception, 183 (55.0%) were aware of emergency contraception and only 69 (37.7%) had used emergency contraception. Lack of adequate knowledge on how to use emergency contraception (63.2%) and fear of side effects (14.9%) were among the reasons for not using emergency contraception (Table 5)*.*

## Discussion

This study was aimed to assess the knowledge, attitude, and practice of contraception methods among female undergraduate students in Dodoma city. We recruited female students of reproductive age between 15 and 49 years with the mean (±SD) age of 27.4 (±5.7). This was an appropriate age limit because the use of contraception is mainly age-dependent. We found the majority (80.4%) of our participants were not married, a finding similar to that from a study among university students in Uganda (87.5%) [16] and another in Kilimanjaro, Tanzania [8], where 76.6% of the participants were also single.

The findings from this study imply being aware of contraception was significantly associated with the age, marital status, and religion of the participants. The reported association is not uncommon, as the marital status and religion of an individual have been shown to influence the awareness and use of contraception among believers [17-19]. The large proportion of participants in this study with knowledge of the methods of contraception is consistent with findings reported elsewhere [5,8,17-18]. It is possible that this high level of knowledge is ascribed to the mass campaign on contraception previously carried out throughout the country.

The majority of participants knew various methods of contraception, with each participant mentioning at least one method. They mostly mentioned condoms (88.0%) and oral contraceptives (83.5%) as the frequently known methods of contraception. However, the overall use of contraceptives was generally low, with only slightly more than half of the students reporting using one or more methods of contraception. Despite condoms and oral contraceptives being the most mentioned methods, their use was relatively low. This finding is consistent with findings previously reported elsewhere [8,10,16,19-20].

The source of information on contraception is key to relaying the correct information to students, particularly the young ones. Our findings show that the most common sources of information were mass media, friends, and health-care workers. Similar findings were reported in studies in Kilimanjaro [8] and Botswana [21] but different from studies among similar groups in India [10], where media was the commonest source of information, and in Nigeria [22], where health care facilities were the most common sources of information. Having a reliable source of information such as mass media and health care facilities/workers is likely to provide youths with more correct and accurate information than friends/relatives; it is thus justifiable to direct efforts toward disseminating information through reliable sources.

Participants showed mixed attitudes toward using contraceptives. Our findings show that the majority of students perceived contraception to be beneficial while a small proportion of students promised to use contraception in the future. On the contrary, about two-thirds agreed that contraception tends to reduce sex drive, with one-third saying condoms can slip off during sexual intercourse. A study in Columbia [23], which aimed to address the impact of oral contraception on libido, observed that most women in the 17 reviewed studies reported an increase in sexual drive when using oral contraceptives. A study in Kilimanjaro [8] reported negative attitudes toward the use of contraception among students because of poor accessibility while participants in a study in Nigeria [24] perceived contraception as a cause of infertility.

Oral contraceptives and condoms were the most preferred methods of contraception among our participants. Our findings were similar to those reported in studies elsewhere [5,7,25]. We further observed that most students would rather prefer to procure contraception from local pharmacies than from health-care facilities due to the long waiting hours associated with other facilities' procedures. This is in line with findings reported in studies elsewhere [7-8]. This practice may imply a lack of sexual and reproductive health services customized to accommodate youths' needs.

Being a more sexually active group, it is not surprising that participants in this study group stated the prevention of sexually transmitted infections as the main reasons for using contraception. The reported reasons are similar to those reported in studies in Ghana [20] and Kilimanjaro [8]. Conversely, a fear of being embarrassed during purchasing and differing religious beliefs were the main reasons for not using contraception. In two studies in Uganda [16,19] religious beliefs were concluded to be key determinants of poor uptake of contraceptives. In this study, one in three non-users claims they do so because their religions condemn the use of contraception. The findings call for necessary measures to address the matter.

About one in three students in this study acknowledged using emergency contraceptives. Lack of knowledge of emergency contraceptives was the main reason given by non-users. The findings are similar to those reported in Ethiopia [26] where about 40% of the students reported using emergency contraception, while in another study done among female undergraduates in Ahmadu Bello University in Nigeria [27], about a quarter of the respondents had used emergency contraception. In Tanzania, for example, individuals who are using emergency contraception are regarded as prostitutes by their peers, a perception that could hinder the use of emergency contraception among students. It is possible that misconception toward the use of emergency contraception between the communities could be the reason for the observed differences.

Limitations

This study has some limitations that need to be taken into account when interpreting the study results. First, the study population consisted of students from one university only. Therefore, we cannot generalize the findings from this study to all female undergraduate students in Tanzania. Second, students' practices and attitudes were self-reported; as a result, there might be an information bias because some information perceived to be sensitive by the students might not be reported. Third, without longitudinal data, it is not possible to construe a true cause and effect relationship between the knowledge and utilization of contraception. Therefore, we cannot draw a predictive conclusion based on these differences.

## Conclusions

Female undergraduate students in this study are aware and have knowledge of contraception; however, their utilization of contraception and emergency contraception is relatively low. Establishing user-friendly reproductive and sexual health services will help improve student uptake of contraception methods.
